# Fresh, Mechanical, and Durability Properties of Self-Compacting Mortar Incorporating Alumina Nanoparticles and Rice Husk Ash

**DOI:** 10.3390/ma14226778

**Published:** 2021-11-10

**Authors:** Bahareh Mehdizadeh, Soheil Jahandari, Kirk Vessalas, Hania Miraki, Haleh Rasekh, Bijan Samali

**Affiliations:** 1 School of Civil and Environmental Engineering, University of Technology Sydney, Sydney, NSW 2007, Australia; bahareh.mehdizadehmiyandehi@student.uts.edu.au (B.M.); kirk.vessalas@uts.edu.au (K.V.); 2 Centre for Infrastructure Engineering, Western Sydney University, Penrith, NSW 2751, Australia; b.samali@westernsydney.edu.au; 3 Department of Civil Engineering, Iran University of Science and Technology, Tehran 6846, Iran; hania_miraki@civileng.iust.ac.ir

**Keywords:** alumina nanoparticles, durability, fresh properties, mechanical properties, rice husk ash, self-compacting mortar

## Abstract

This paper presents a comprehensive evaluation on self-compacting (SC) mortars incorporating 0, 1, 3, and 5% alumina nanoparticles (NA) as well as 0% and 30% rice husk ash (RHA) used as Portland cement replacement. To evaluate the workability, mechanical, and durability performance of SC mortars incorporating NA and RHA, the fresh properties (slump flow diameter and V-funnel flow time), hardened properties (compressive strength, flexural strength, and ultrasonic pulse velocity), and durability properties (water absorption, rapid chloride permeability, and electrical resistivity) were determined. The results indicated that the addition of NA and RHA has negligible effect on the workability and water absorption rate of the SC mortars. However, significant compressive and flexural strength development was observed in the SC mortars treated with NA or the combination of NA and RHA. The introduction of RHA and NA also reduced the rapid chloride permeability and enhanced the electrical resistivity of the SC mortars significantly. It is concluded that the coexistence of 30% RHA and 3% NA as cement replacement in SC mortars can provide the best mechanical and durability performance.

## 1. Introduction

Self-compacting concrete (SCC) technology was introduced by Okamura in 1986 [1]. Over the recent decades, the technology of SCC has been an attractive option for industry practitioners worldwide due to its various benefits [1,2,3,4,5,6]. Enhancing the properties of cement paste and mortar has been the key focus of various research studies conducted in the past. Domone and Jin [7] reported three main advantages for investigating SCC technologies in mortars: (1) the amount of aggregates in SCC (usually 31–35% by volume) is lower compared to normal concrete, and thus the mortar characteristics govern the properties of SCC, (2) the mortar properties are among the main controlling factors in the SCC mix design procedures, and (3) the procedure of testing mortars compared to concrete is more convenient.

The increased application of pozzolans as supplementary cementitious material (SCM) additions has been growing as they can improve the mechanical and durability performance of concrete and/or mortar [8,9,10,11,12,13,14,15,16]. Former studies have revealed the effective contribution of fly ash as a pozzolanic by-product in concrete and/or mortar by reducing the cement consumption and improving the performance of cement paste and mortar [6,17,18,19,20,21,22,23].

Rice husk is known as one of the main agricultural by-products attained during the milling process of rice grains to remove the husk. Rice husk is a sustainable material and is available in large quantities in rice-producing countries. Rice husk ash (RHA) can be effective as SCM alternative because of its pozzolanic properties [18] due predominantly to (a) its high surface area, (b) high silica content, (c) high distorted crystallization presence of amorphous silica, and (d) the reduced particle size resulting from the process of grinding [24]. The use of RHA as a SCM can effectively develop the mechanical properties of cement mortars, such as their compressive and flexural strengths [19]. The other advantage of partially replacing cement with RHA for SCM use is the substantial reduction in carbon dioxide emissions and concrete production costs that follows due to less Portland cement use and more waste material utilization.

Using nanoparticle technologies, with the aim of producing more economical concrete, is also a practical approach to produce concrete with standard properties, primarily when superplasticizers (SP) and additional admixtures are used to produce SCC. Several researchers around the world have examined the effects of using nanoparticles in concrete; however, limited studies have focused on the effectiveness of using alumina nanoparticles (NA) in SCC. Oltulu et al. and other researchers [25,26,27] studied the capillary permeability as well as compressive strength development of cement mortar containing NA and silica fume. They concluded that with partial replacement of cement by NA, the compressive strength of concrete is improved while the workability is reduced. The findings revealed that the incorporation of NA also enhances the split tensile strength of cement mortar. The optimum strength in the cement mortar was achieved when 1% (by weight) NA was introduced. Leon et al. [28] investigated the effectiveness of using NA in cement mortar as a waste material from agriculture production facilities.

In the current study, the effect of using both RHA and NA on the fresh, mechanical and durability properties of self-compacting (SC) mortars are investigated. According to the previous studies, in the binary mortars containing nanomaterials and RHA, 0–30% of RHA have been introduced as an effective range to improve the mechanical behaviour and durability properties of specimens [20,29,30].

This is novel work as utilizing both RHA and NA as ternary blend with ordinary Portland cement (OPC) not been studied before. Multiple contents of RHA and various percentages of NA are employed as partial substitutions of OPC. A series of parameters such as compressive strength, electrical resistivity, water absorption, rapid chloride permeability, etc., are investigated to evaluate the effects of RHA and NA on SC mortars. Moreover, previous studies have not proposed any optimal mix design when a ternary blend of RHA, NA, and ordinary Portland cement are employed, while this study has proposed an optimal mix design considering the fresh and hardened properties of SC mortars with positive environmental credentials.

## 2. Experimental Program

### 2.1. Materials

River sand with a maximum grain size of 1.18 mm, specific gravity of 2.45, and 24-h water absorption rate of 2.38% was used as a fine aggregate in the SC mortars in this study. Ordinary Portland cement (OPC) Type II following ASTM C778 [31] and ASTM C150 [32] was sourced from Momtazan Cement Factory in Kerman, Iran. About 91% of the total mass of the used RHA is silica. This composition represents that potential that it can be employed as a pozzolanic material in cement mortar according to ASTM C311 [33]. The physical properties and chemical composition of RHA and OPC are summarized in Table 1. A poly-carboxylate type high-range water-reducing admixture (SP) with a density of 1.03 g/cm^3^ meeting the requirements of ASTM C494 [34] was used to obtain a reasonable workability and better dispersion of the NA. The SP content was determined through trial tests to maintain and reproduce a similar workability level in all the mortar mixes. NA was also incorporated into the mixes in the form of an aqueous solution. The chemical composition of NA is reported in Table 2. Previous research studies have stated that the quality of water can also affect the properties of concrete and cementitious materials [5,35,36,37,38,39,40,41,42]. However, in this research, distilled and tap water were respectively used for the characterization tests and for sample preparation studies according to the recommendation of some previous studies [43,44,45,46,47,48,49,50,51,52].

### 2.2. Mix Proportions

Eight different mixes were designed and prepared to study the properties of the SC mortars. RHA was replaced with Portland cement at ratios of 0% and 30% and various percentages of NA (including 0, 1, 3 and 5% (by weight)) were replaced with the cumulative amount of Portland cement and RHA. The ratio of water/binder was considered as 0.40 in all the mixes. Poly-carboxylate type SP at percentages of 0.62% by weight of the binder was used to maintain the slump flow diameter of the fresh mortar between 240 mm and 260 mm. Various amounts of sand was utilized to obtain a reasonable and reproducible slump flow diameter in the mortars with different proportions of OPC, RHA, and NA. The abbreviations used for labelling in this study were adopted in such a way that the numbers before RHA and NA stand for the percentages of RHA and NA, respectively. For example, 30RHA5NA represents partial substitution of OPC in SC mortars with 30% RHA and 5% NA. CO represents the control specimen incorporating OPC, water, sand, and SP without any RHA and NA. Table 3 presents the mix proportions of the mortars.

### 2.3. Sample Preparation

The ASTM C305 [53] method was followed in this study as a guide for sample preparation. The potential toxicity of nanoparticles has recently raised some concerns among researchers despite NA been used in many research projects [54]. Using PPE including masks and gloves during the mortar mixing is mandatory to prevent any direct contact with the NA.

After some preliminary tests, OPC and RHA were dry mixed thoroughly for 60 s with medium velocity (80 rpm). Then, 30% of water with NA and RHA were added to the dry mix. Later, the 70% water remainder and SP were blended, added to the mix and stirred for 30 s at normal speed (75 rpm). The mixing of fresh mortar was paused for 90 s and then continued for 60 s at high speed (100 rpm) to achieve a mortar with constant NA distribution. The fresh mortar was then poured into two types of moulds: first, 50 mm × 50 mm × 50 mm cubic moulds and second, 50 mm × 50 mm × 200 mm prismatic moulds, for the various types of tests. The curing was conducted by placing the samples in lime-saturated water with a temperature of 23 ± 2 °C for different curing duration before testing.

### 2.4. Testing of Specimens

The fresh properties of prepared mortars were determined via standard of slump flow diameter of 250 ± 10 mm and mini V-funnel flow time tests as recommended by EFNARC [55]. The mentioned tests were conducted due to the fact that the results of the slump flow diameter and mini-V-funnel tests are tied to the yield stress and plastic viscosity, respectively. Any separation or bleeding of the mortars was observed visually. These tests were conducted to observe, when the mortars were fully combined for sample preparation, based on the following procedure: sand and OPC were blended at a speed of 80 rpm for about 60 s. Then the NA powder, the binder and RHA, as well as the 30% of the water were well blended for around 60 s more. The obtained mortar mix was then rested for 90 s. The high-range water-reducing SP and the remaining water (70%) were eventually introduced and completely combined for 120 s.

The mechanical and durability properties of the hardened mortar were determined and evaluated by performing comprehensive experimental tests including compressive strength, flexural strength, electrical resistivity, water absorption, and rapid chloride permeability test (RCPT).

The compressive and flexural strengths of the cured samples were determined at 7, 28, and 90 days and the water absorption were measured at 28 days. Furthermore, the electrical resistivity was measured at 28 and 90 days, and the RCPT was conducted at 90 days.

To study the mortar’s water absorption, the samples were initially placed in an oven at a temperature of 105 ± 5 °C for 24 h, followed by measuring their oven-dry mass (Wd). Afterwards, the samples were submerged in water for 24 h, and once again, their mass was measured (Ws). Eventually, the percentage of water absorption was calculated as follows:

(1) $$\mathrm{Water}\;\mathrm{absorption}\;\left(\%\right)\;=\frac{\mathrm{Ws}-\mathrm{Wd}}{\mathrm{Wd}}\times 100$$

The procedure for water absorption test including the exposure of specimens to the temperature of 105 ± 5 °C was in accordance with ASTM C642 [50]. Additionally, a comprehensive experimental study conducted by the authors has indicated that such a temperature does not significantly affect the water absorption rate or microstructure of the samples [56].

According to the ASTM C305 [53], three replicate samples were tested for each condition to ensure the results accuracy, and their average values were reported as the representative. In total, 72 samples were tested to measure the compressive strength and 72 samples to measure the flexural strength of the samples cured for 7, 28, and 90 days. In addition, 24 samples were tested for the water absorption after 28 days of curing. Moreover, 48 samples were tested to measure the electrical resistivity at 28 and 90 days of curing. Finally, 24 samples were tested for the RCPT after 90 days of curing. In total, 240 tests were conducted to measure the different properties of the specimens.

The electrical resistivity test was carried out due to the fact that the electrical properties of concrete is tied to the porous microstructure, permeability, ion transport, and moisture content of the concrete. Electrical resistivity can also be utilized to evaluate corrosion potentials in reinforced concrete. Corrosion is a complex electrical-chemical process that oxidizes metal in virtue of the current flow. For the electrical resistivity test, 50 mm × 50 mm × 50 mm cube samples were cured and used at 28 and 90 days to reduce the risk to corrosion. Two copper plates were adhered to the lower and upper surfaces of the mortar samples by the cement paste. This step was carried out after drying the specimen surface and prior to placing two wooden nano-particle blocks on these plates. The resistivity test entailed electrical resistance in which two electrodes were linked to both sides of the samples. The electrical resistivity value (p) in kΩ·cm was measured as follows:

(2) $$p=\frac{RA}{L}$$

where *R* signifies the resistance (Ω), L denotes the length (mm) of the sample and *A* is a sign of the sample cross-sectional area (mm^2^).

The rapid chloride permeability test (RCPT) is also considered a further approach to evaluate the durability properties of the hardened concrete or mortar. According to ASTM C-1202 [57], cylindrical specimens are cut to cylinders with a length and diameter of 100 and 50 mm, respectively. Subsequently a sequence of transmission charges can be sampled and acquired on a computer for about 6 h. It should be mentioned that the results of this test only demonstrate the performance of the samples against chloride permeability and they do not present a direct measurement of permeability. In general, lower contents of chloride ions represent more desirable durability properties achieved in the mortar.

## 3. Results and Discussion

### 3.1. Fresh Properties

Table 4 shows the slump flow diameters and V-funnel flow times of the SC mortars. The range of slump flow diameters is between 240 and 255 mm while the range of the V-funnel flow times fall between 8.3 to 11 s. These slump flow diameters and V-funnel flow times reveal that the highest workability levels are achieved with the samples containing 5% NA and 30% RHA. Moreover, the SC mortars containing both RHA and NA obtained higher workability levels than the SC mortars comprising only NA or the control SC mortar devoid of NA and RHA. A comparison between the slump flow diameters and V-funnel flow times of SC mortars with and without RHA (containing similar NA contents) also indicates that SC mortars with RHA are slightly more workable than the SC mortars without RHA. This could be due to the lower amounts of sand in the SC mortars containing RHA that resulted in a slightly higher workability level. In other words, the amount of solid materials in the mixture affect the workability and the amount of free water in the mixture; such that when the amount of solid materials decrease, the free water in the mixture increases, which brings about higher workability level. Similar results have been reported in previous studies as well [58].

### 3.2. Hardened Properties of SCMs

#### 3.2.1. Compressive Strength

**Figure 1:**
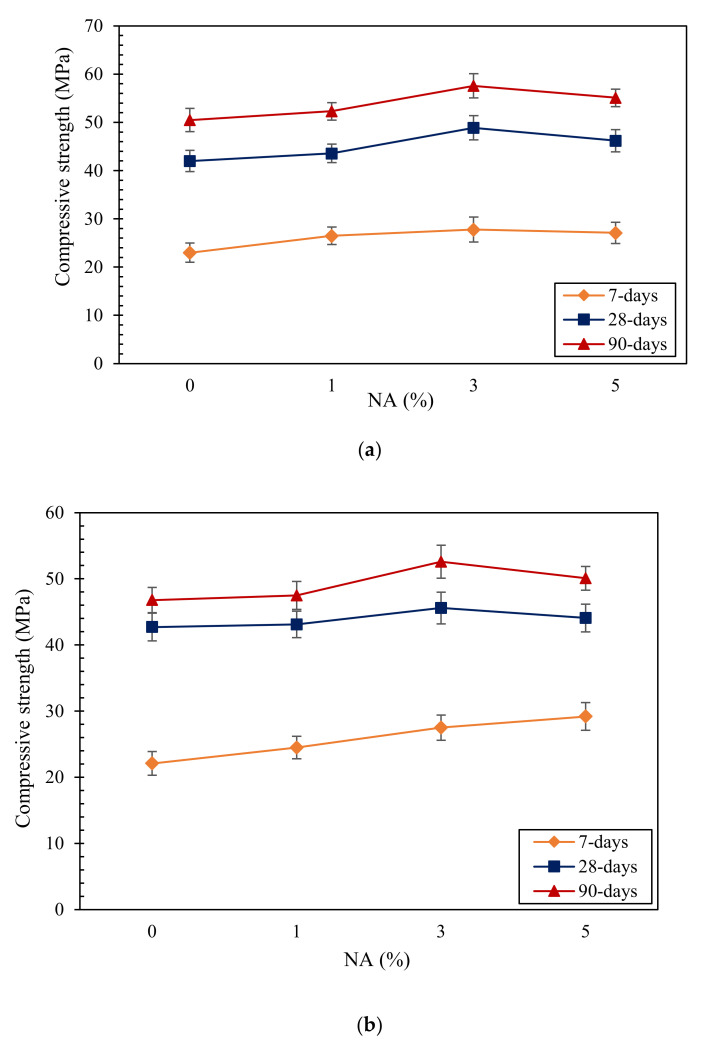
Compressive strength of SC mortars, (**a**) RHA = 0%, (**b**) RHA = 30%.

The outcomes of compressive strength tests are presented in Figure 1. Obviously, by increasing the curing time, the compressive strength of all samples improved, irrespective of the used additives. As shown in Figure 1, there appears to be an added benefit using NA in SC mortars. Samples with 3% NA had the highest value of compressive strength among all the samples with the compressive strength values of 27.8, 48.9, and 57.5 MPa after 7, 28, and 90 days of curing, respectively. The compressive strength of specimens with both Na and RHA were greater than the control sample. This can be explained by the ability of NA and RHA to fill the pores in mortars, which improves the compressive strength of samples. Adding 5% NA to samples negatively affected the compressive strength of samples compared with those including 3% NA. This could be due to the impact of an excessive amount of NA which reduced the hydration rate of mortar samples and resulted in the compressive strength reduction. If the amount of NA and the distance available between these nanoparticles is suitable, the growth of the Ca(OH)_2_ crystals become restricted. As a result, the rate of growth of the Ca(OH)_2_ crystals will be controlled. From another perspective, NA plays a pivotal role as an activating agent to hasten cement hydration due to its higher state of free energy. Comparing the modified samples with the control sample, all modified samples showed higher compressive strength values at various curing ages except for 30RHA. Therefore, the coexistence of RHA and NA could improve the compressive strength of mortar samples. Oltulu et al. [25] recommended the use of 1.25% NA powder as the optimum content to improve the capillary permeability and compressive strength of cement mortar. Rashad et al. [26] also observed a similar relationship with an enhancement in the compressive strength of concrete samples by partially substituting cement with NA.

#### 3.2.2. Flexural Strength

**Figure 2:**
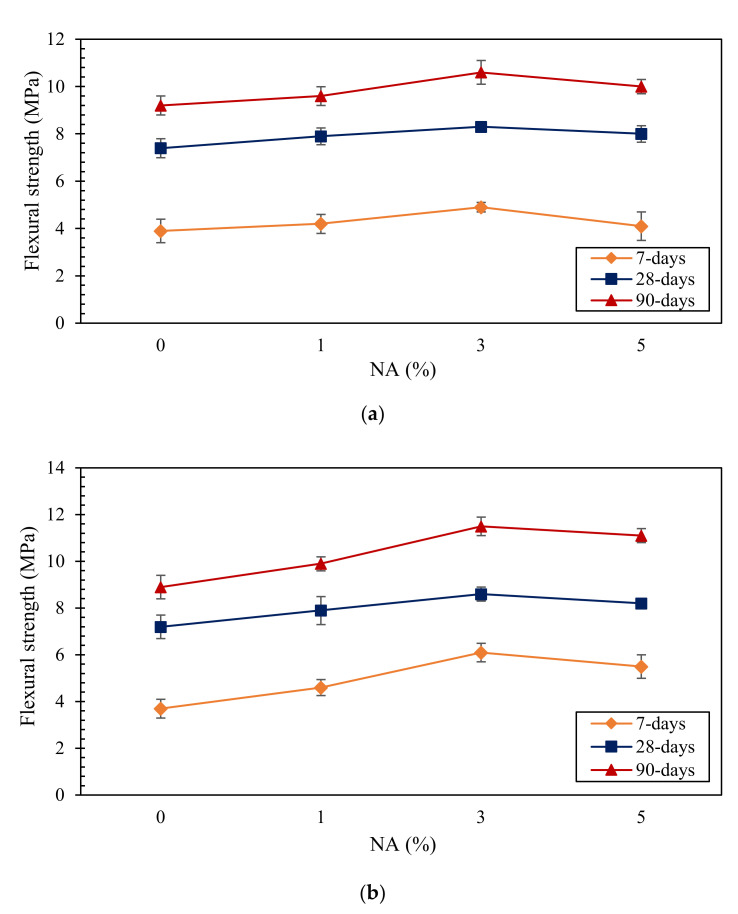
Flexural strength of SC mortars, (**a**) RHA = 0%, (**b**) RHA = 30%.

The flexural strength values of the SC mortars at different curing ages are shown in Figure 2. All samples exhibited increasing flexural strength by increasing the curing period. The 30RHA3NA sample set, with flexural strength values of 6.1, 8.6, and 11.5 MPa after 7, 28, and 90 days, respectively, indicated the highest flexural strength among all samples. As shown in Figure 2, a decrease in the flexural strength was apparent when the amount of NA exceeded 3%. All results showed an enhancement in the flexural strength of modified samples in comparison to the control sample. The reason could be on the account of the rapid consumption of Ca(OH)_2_ at early ages during cement hydration. All the treated samples, except 30RHA, had higher flexural strength values at the ages of 7, 28, and 90 days. This proves the positive impact of the simultaneous use of RHA and NA in the SC mortars to improve their mechanical properties.

#### 3.2.3. Water Absorption

**Figure 3:**
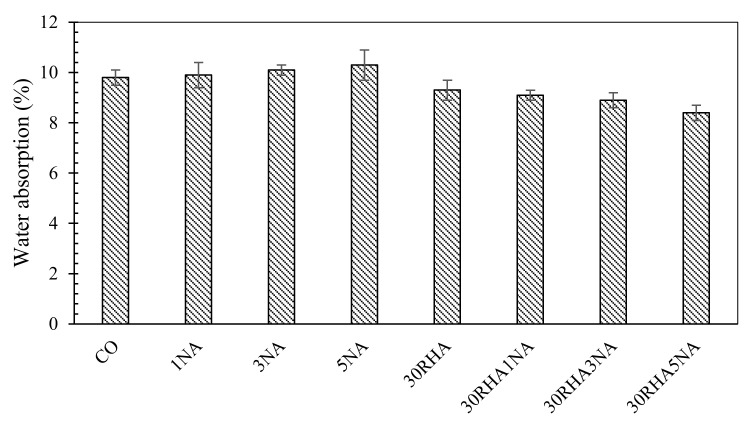
Water absorption rate of SC mortars.

The water absorption values of 90-day cured SC mortars are presented in Figure 3. Mixes without RHA addition are observed to slightly increase their water absorption by the addition of NA. Zhang et al. [59] also reported that increasing NA content leads to a coarser pore structure in concrete. It appears that an increase in the NA content reduces the pore structure refinement of the mortar. This could be attributed to the space limitations on the account of the reduction in the distance between NA present in higher concentration. Thus, the formation of calcium hydroxide crystals becomes limited. As a result, the proportion of crystals becomes lowered compared with the amount of C-S-H gel and the water absorption increases. The water absorption enhancement noted in samples with a comparatively high NA content is also due to the dilution effect and the lower amount of OPC that is present. A lower amount of OPC decreases the amount of hydration products in the system.

Incorporation of the RHA and NA as partial cement replacement in SC mortars also resulted in a reduction in the water absorption. The most substantial reduction was observed in 30RHA5NA, which exhibited an almost 14% decrease in the water absorption compared to the control sample. This improvement signifies an improvement in achieving a more impermeable mortar. The noted water absorption decrease could be tied to the impact of pozzolanic reactions through which Ca(OH)_2_ is consumed, and more C-S-H is generated, resulting in a more compact and densified microstructure.

In general, the positive impact of the incorporation of pozzolanic materials, including NA and RHA, on the characteristics of water absorption, could be attributed to: (1) the pozzolanic material (RHA) plays a role as a filler, which reduces the pore size and brings about less permeability in the paste [59,60], and (2) the enhancement of the interfacial transition zone (ITZ) in mortars on account of the pozzolanic reaction and additional C-S-H formed from both NA and RHA presence in these systems. However, the pore structure is likely due to the NA also reacting with Ca(OH)_2_ to form secondary C-A-S-H phases, which are also generated during cement hydration. As a result, the cement hydration is expedited as the RHA and NA both contribute to reducing the volume of the large-sized pores. Zhang et al. [61,62] also reported an improvement in the performance of the ITZ by the incorporation of nanofillers. Torkaman et al. [63] also reported a reduction in the water absorption rate of concrete specimens as an indication of durability due to addition of RHA.

#### 3.2.4. Electrical Resistivity of SC Mortars

**Figure 4:**
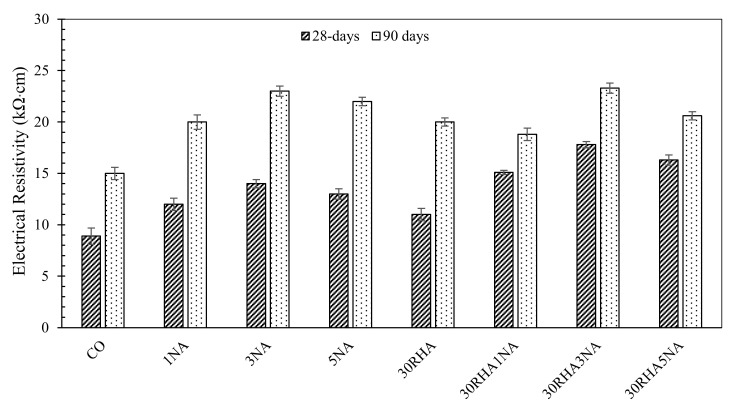
Electrical resistivity (kΩ·cm) of SC mortars at different ages.

Table 5 presents the correlation between electrical resistivity and corrosion rate of SC mortars. The outcomes of the classification categories of electrical resistivity aligning to probability rate for various mixes are presented in Figure 4.

According to Table 5, the risk of corrosion for all samples is low to moderate. At 28 days, the sample with 3% NA and 30% RHA had the lowest risk to corrosion (17.8 kΩ·cm), which means the probability of corrosion is low. In samples cured for 90 days, the corrosion probability for all mixes was low and had up to 85% improvement compared to the control sample. As the electrical resistivity is one of the critical parameters governing the initiation and propagation of steel reinforcement corrosion [64], using 30% RHA and 3% NA in mortar is recommended as an effective option to enhance the durability of SC mortars.

#### 3.2.5. Rapid Chloride Permeability Test (RCPT)

Determining the chloride permeability of the mortars is a valuable technique to investigate the durability of SC mortars incorporating RHA and NA. To date, several studies have focused on evaluating the durability of concrete and mortars by the RCPT [60,64]. Corrosion of steel reinforcement is one of the most important durability concerns in field concrete structures. Even though absorption is the principal mechanism of chloride migration for the near-surface unsaturated hardened concrete, the accumulation of chlorides in this layer is due to the further penetration of chlorides into the concrete through diffusion [65,66]. One of the approaches for assessing the quality of concrete or hardened mortar is the RCPT method. Additionally, the RCPT method does not have a direct relationship to the reinforcement corrosion rate, it can directly evaluate the relative resistance of the concrete to the penetration of chloride ions [66,67]. Joshaghani et al. [68] found out that with increasing the level of electrical resistivity, the RCPT value decreases and the relationship between the electrical resistivity and RCPT indicates a high correlation. The RCPT results confirm that with the addition of nanoparticles, the penetration rate of chloride ions decreases. These reductions are attributed to the ability of the nanoparticles to densify the microstructure of the mix by filling the nanopores and to improve the hydration process.

**Figure 5:**
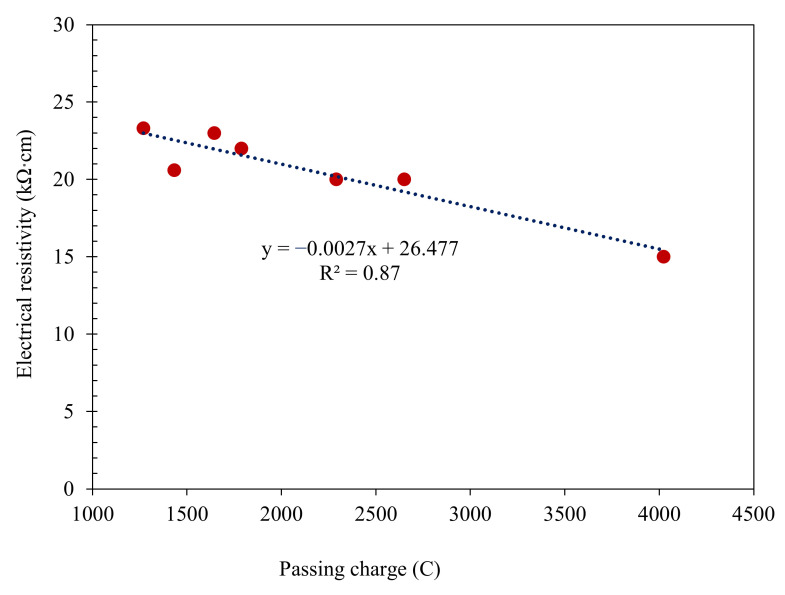
Statistical correlation between electrical resistivity and RCPT at 90 days curing for SC mortars.

The values of chloride permeability applied to SC mortars are outlined in Table 6. Using NA was observed to decrease the chloride permeability of the SC mortars. The findings demonstrate that the control sample had the highest value of chloride permeability, rated as “moderate,” with a cumulative charge passed of 3800 Coulombs. The introduction of NA in the SC mortars brought about a decrease in the samples’ chloride permeability, which were classified as “low” except for 1NA and 30RHA. The lowest chloride permeability value was obtained for the combined 30% RHA and 3% NA mix. This reduction in chloride permeability can be regarded due to the denser microstructure and a decrease and discontinuity of the pores. Furthermore, a valid statistical correlation (R^2^ = 0.87) was obtained between the rapid chloride permeability test and the electrical resistivity at 90 days of curing for the mixes. These results are presented in Figure 5. By increasing the chloride permeability of specimens, the electrical resistivity decreases. Tabarelli et al. [69] also reported a significant reduction in the chloride diffusion coefficient by increasing the curing time from 3 to 7 and 28 days which proves the fact that prolonged curing can decrease the risk of corrosion in reinforced concrete structures.

## 4. Conclusions

This research consisted of a series of experiments to investigate both the single and combined effect of incorporating alumina nanoparticles (NA) and rice husk ash (RHA) on the fresh, mechanical and durability properties of self-compacting (SC) mortars. According to the findings of slump flow diameter, V-funnel flow time, compressive strength, flexural strength, water absorption, ultrasonic pulse velocity, rapid chloride permeability, and electrical resistivity tests, the main conclusions are summarized as follows:

1. Although adding NA contributed to subtle changes in the fresh properties of SC mortars, the introduction of RHA brought about higher workability.
2. The replacement of cement with 30% RHA led to a 13% reduction in the compressive strength of SC mortars. The compressive strength of mortars containing RHA tended to increase by using higher contents of NA. The most effective content of NA for increasing the compressive strength in the mortars was found to be 3% by weight of the binder.
3. Compressive and flexural strengths of SC mortars improved significantly by the introduction of NA and RHA to the mixes because of further C-S-H gel formation. The best result was achieved when 3% NA and 30% RHA was used. When the percentage of NA exceeded 3%, a decrease in the strength was observed owing to the excessive amount of NA.
4. The water absorption of SC mortars without RHA increased as the NA increment added increased; however, with the introduction of RHA, as the amount of NA increased, the water absorption decreased, which was due to the denser structure of the SC mortars.
5. The results of electrical resistivity indicate low to moderate risk of corrosion for all 28-day cured samples. At 28 days, the sample with 3% NA and 30% RHA had the optimum amount of electrical resistivity. After 90 days of curing, the corrosion probability for all SC mortars had a lower rate. The results confirm the formation of condensed and densified mortar.
6. Using NA decreased the probability of corrosion. Nonetheless, when RHA was added, the results were enhanced as the probability for corrosion was further reduced. The best result was obtained for the SC mortar compromising a combination of 3% NA and 30% RHA.
7. Considering the improved fresh, mechanical and durability properties of SC mortars, it is recommended to replace 30% and 3% of cement with RHA and NA, respectively.

It could be perceived that the investigated mixtures in this study are more cost-effective compared to the commercial SC since Portland cement has been replaced with RHA. Moreover, the outcome of this research indicated the desirable mechanical and durability properties of the studied mixtures. Hence, lower cost and desirable performance of these mixtures account for its privilege compared with commercial SC. However, a cost analysis is recommended to prove these statements.

## Figures and Tables

**Table 1:** Physical characteristics and oxide composition of RHA and OPC [31,32].

	Constituents	OPC (wt.%)	RHA (wt.%)
**Chemical composition**	SiO_2_	21.75	91.15
	Al_2_O_3_	5.15	0.41
	Fe_2_O_3_	3.23	0.21
	CaO	63.75	0.41
	MgO	1.15	0.45
	SO^3^	1.95	0.62
	KO	0.56	6.25
	Na_2_O	0.33	0.05
	L.O.I	2.08	0.45
**Physical properties**	Surface area (m^2^/g)	0.31	4.09
	Specific gravity (g/cm^3^)	3.15	2.07

**Table 2:** NA properties [33].

Nanoparticles	Average Diameter (nm)	Specific Surface Area (m^2^/g)	Purity (%)
Al_2_O_3_	15 ± 3	200	>98

**Table 3:** Mix design of mortars.

Sample ID	OPC (kg/m^3^)	RHA (kg/m^3^)	NA (kg/m^3^)	Water (kg/m^3^)	Sand (kg/m^3^)	SP (kg/m^3^)
CO	700	0	0	280	1266	4.3
1NA	693	0	7	280	1253	4.3
3NA	679	0	21	280	1228	4.3
5NA	665	0	35	280	1202	4.3
30RHA	490	210	0	280	1027	4.3
30RHA1NA	483	210	7	280	1182	4.3
30RHA3NA	469	210	21	280	989	4.3
30RHA5NA	455	210	35	280	963	4.3

**Table 4:** Fresh properties of SC mortars.

Sample ID	Slump Flow Diameter (mm)	V-Funnel Flow Time (s)
CO	240	11.0
1NA	242	10.5
3NA	245	10.5
5NA	248	9.5
30RHA	255	8.3
30RHA1NA	255	8.6
30RHA3NA	252	8.9
30RHA5NA	250	9.4

**Table 5:** Correlation between electrical resistivity and corrosion rate [35].

Electrical Resistivity (KΩ·cm)	Rate of Corrosion Risk
>20	Low
10–20	Low to moderate
5–10	High
<5	Very high

**Table 6:** Results of rapid chloride ion permeability test (RCPT) for SC mortars.

Sample ID	Passing Charge (C)	Permeability Class (ASTM C1202 [28])
CO	4023	High
1NA	2290	Moderate
3NA	1645	Low
5NA	1788	Low
30RHA	2650	Moderate
30RHA1NA	1487	Low
30RHA3NA	1269	Low
30RHA5NA	1433	Low

## Data Availability

Data is contained within the article.
